# Mr. Rodman, on the Influenza

**Published:** 1803-07-01

**Authors:** John Rodman

**Affiliations:** Paisley


### *To the Editors of the Medical and Physical Journal.*

Gentlemen,

Agreeably to your request in No. 51, which, if properly attended to, may prove to be considerably advantageous in forming just opinions respecting Influenza, I send you the following, for insertion in your very useful Journal.

This disease, which has attracted so much notice as the reigning epidemic, and to which the name of Influenza has been given, appeared in Paisley about the beginning of last April, became very general in a few weeks, and was distinctly marked by the characteristic symptoms so well described by several authors. The weather, both before and since that time, has been particularly cold and variable.

By the middle of May this disease was considerably abated in frequency, and at present there are but few afflicted with it. Upon the whole, the Influenza has been very mild, and so far as I can learn, not above two have died of it in this town.

The greatest number who were affected with it could go about their ordinary business, and most of those that were ill and took to bed, recovered in four or five days. Many indeed were longer confined, but it appeared invariably, to be either the consequence of inattention or former disease.

In a daily statement of the cases of fifty-eight patients, who had Influenza pretty severely, taken simply as they occurred, and at a time that the disease was not prevalent, there are twenty-six males and thirty-two females. The whole of these recovered within eight days of the commencement of the disease, excepting three, a man and two boys, who were from ten to fourteen days ill before the feverish symptoms disappeared; but these three were in a diseased state previous to the attack of Influenza.

Among

Among other families which I had occasion to see, when part was under this disease, there were twenty-seven, in which every individual of the families were less or more affected. In these families, nine children only were at the breast, none of them younger than a month, and they all had the disease soon after, not before, the mother. One family, consisting of six, was under the disease for several weeks, and the mother and two daughters, who were first affected, took it a second time while attending the other three. In another family the mother became ill of Influenza and soon recovered, but before the whole had passed through the disease, she again took it, two weeks after her recovery, and was considerably worse than at first.

As proper attention to acid fumigations is not easily procured, without being more frequently with the family than ordinary practice in a town will allow of, I wish not to draw any conclusion from what experiments of that kind I have made. However, if justified in choosing the least of two evils, one of which is unavoidable, we certainly act with propriety when we admit that Influenza is infectious, till clearer facts convince us that it is not. By doing so we the more readily attend to every mean of prevention, and give a chance, at least, of keeping off disease without any bad effect; whereas, by inculcating a contrary opinion, which is very disputable, we may propagate an error that may lead to serious consequences. Any argument brought forward by the opponents to infection seems feeble, when compared to the many instances which could be given to the contrary. That typhus fever comes on in persons who are not exposed to that infection, so far as it is possible to ascertain, can be proved in many cases, and who, from such a circumstance, would doubt that typhus is contagious. The short view of cases of individuals and families stated above, I rather advanced, than others of a similar nature to which I was less attentive, not having marked them down as they occurred. If they tend not to confirm the opinion that Influenza is infectious, they are facts which, in some measure, increase the difficulty of opposing it. As to blood-letting in this disease, which has been treated with so much invective by some authors, it was both necessary and useful in a few cases. For my own part I had no occasion to use the lancet, but would I say from that, that in no case such a thing was necessary, when I know some practitioners in town, of long experience and considerable abilities, who were were compelled from the violence of symptoms, to use the lancet, and had the happiness to see its good effects.

People affected with Influenza, like catarrh, seemed more than usually liable to suffer from cold air; and though not exposed to cold in any manner different to what they were accustomed, yet the being without doors occasionally, was frequently the cause of increased disease, and of course inflammatory symptoms; thus, from predisposition, and other circumstances, was blood-letting rendered necessary. Another cause of increasing these symptoms, which deserves particular attention and severe censure, was the noted cure recommended so highly in the public papers, viz. whisky and oatmeal. It is to be regretted that whisky drinking is so much the pleasure of many; and in consequence of this recommended cure, numbers considered themselves as having got a licence to drink plenty of whisky; of course, there was not only pretended affection, but even when it was real, whisky was drank greedily, till severe illness changed their inclinations, and obliged them to apply for remedies more suited to their complaints.

Considering it not necessary to extend this paper by commenting at large on the mode of cure,

I remain, &c.

*Paisley, June* 7, 1803. JOHN RODMAN.

