# Erratum

**Published:** 1803-08-01

**Authors:** 


### ERRATUM.

Vol. x. p. 46, l. 7, from bottom, for *not*, read *most*.

